# Dynamic surface topography data for assessing intra- and interindividual variation of vertebral motion

**DOI:** 10.1016/j.dib.2023.109178

**Published:** 2023-04-26

**Authors:** Martin Haimerl, Alina Linkerhägner, Jürgen Konradi, Claudia Wolf, Philipp Drees, Ulrich Betz

**Affiliations:** a Innovation and Research Center Tuttlingen, Furtwangen University of Applied Science, Kronenstraße 16, 78532 Tuttlingen, Germany; b Institute of Physical Therapy, Prevention and Rehabilitation, University Medical Center of the Johannes Gutenberg University Mainz, Langenbeckstr.1, 55131 Mainz, Germany; c Department of Orthopedics and Trauma Surgery, University Medical Center of the Johannes Gutenberg University Mainz, Langenbeckstr.1, 55131 Mainz, Germany

**Keywords:** Motion analysis, Human spine, Spinal kinematics, Gait analysis, Intra- and inter-individual variation, Surface topography, Rasterstereography

## Abstract

Spinal function is substantially related to the motion of the particular vertebrae and the spine as a whole. For systematic assessment of individual motion, data sets are required which cover the kinematics comprehensively. Additionally, the data should enable a comparison of inter- and intraindividual variation of vertebral orientation in dedicated motion tasks like gait. For this purpose, this article provides surface topography (ST) data which were acquired while the individual test persons were walking on a treadmill at three different speed levels (2 km/h, 3 km/h, 4 km/h). In each recording, ten full walking cycles were included per test case to enable a detailed analysis of motion patterns. The provided data reflects asymptomatic and pain-free volunteers. Each data set contains the vertebral orientation in all three motion directions for the vertebra prominens down to L4 as well as the pelvis. Additionally, spinal parameters like balance, slope, and lordosis / kyphosis parameters as well as an assignment of the motion data to single gait cycles are included. The complete raw data set without any preprocessing is provided. This allows to apply a broad range of further signal processing and evaluation steps in order to identify characteristic motion patterns as well as intra- and inter-individual variation of vertebral motion.

**Specifications Table**

**Table:** 

Subject	Health and medical science
Specific subject area	Orthopaedics, Sports Medicine and Rehabilitation
Type of data	Excel file csv files
How the data were acquired	The Motion data were acquired using the surface topography system DIERS 4D motion® Lab / DIERS formetric 4D system (Version 3.10.1, Diers International GmbH, Schlangenbad, Germany) in combination with a treadmill and the pedogait component. The treadmill was used for performing gait sequences at a fixed speed level. The pedogait component is a dynamic foot pressure measurement unit which was utilized for identifying the particular steps / gait cycles.
Data format	Raw
Description of data collection	3d vertebral orientation data from VP down to L4 as well as pelvis were acquired when the test persons were walking at three different speed levels (including ten full gait cycles per data set). The acquisition time for each test sequence was approximately 20 seconds. The sampling frequency of the surface topography system was 60 Hz. No normalization of step length was performed.
Data source location	Diers International GmbH, Schlangenbad, Germany
Data accessibility	Repository name: Mendeley Data Data identification number: doi: 10.17632/y8nwmwvs5n.1 Direct URL to data: https://data.mendeley.com/datasets/y8nwmwvs5n/1 Full citation of data set: Haimerl, Martin; Konradi, Jürgen; Wolf, Claudia; Nebel, Iman; Linkerhaegner, Alina; Drees, Philipp; Betz, Ulrich (2022), “Dynamic Surface Topography Data for Assessing Intra- and Interindividual Variation of Vertebral Motion”, Mendeley Data, V1, https://doi.org/10.17632/y8nwmwvs5n.1
Related research article	M. Haimerl, I. Nebel, A. Linkerhägner, J. Konradi, C. Wolf, P. Drees, U. Betz. Comprehensive visualization of spinal motion in gait sequences based on surface topography. Hum Mov Sci. (2022) 81:102919. https://doi.org/10.1016/j.humov.2021.102919 In between, the following additional publication refers to the data: M. Haimerl, I. Nebel, A. Linkerhägner, J. Konradi, C. Wolf, P. Drees, U. Betz. Consistency of vertebral motion and individual characteristics in gait sequences. Human Movement Science. 2023 Feb;87: 103036. https://doi.org/10.1016/j.humov.2022.103036

## Value of the Data

• Data can be used to assess intra- and interindividual variation of vertebral motion in gait sequences where asymptomatic and pain-free test persons are walking at different speed levels.
• The inclusion of ten gait cycles (representing the system's maximum processing capacity) allows a more detailed analysis of variation between gait cycles and provides better options for signal processing, e.g. filtering / extracting frequencies of basic gait cycles.
• Data can be used by researchers in motion analysis to figure out characteristic motion patterns and variation of motion sequences.
• The full set of raw data is provided to allow a broad range of signal processing and evaluation steps for comparing intra- and interindividual variation of spinal motion.

## 1 Objective

An analysis of intra- and interindividual variation is a key element for identifying personal characteristics in motion sequences. Without high in-person consistency, a motion feature is not able to characterize individual behavior. In this paper, data is provided which enables such a comparison for vertebral motion in gait sequences. For this purpose, dynamic surface topography (ST) data was acquired for a set of twelve asymptomatic and pain free volunteers that walked on a treadmill at different speed levels (2 km/h, 3 km/h, and 4 km/h). ST was chosen as a measurement technique since it allows a comprehensive recording of motion in all three dimensions across a wide range of vertebrae. Additionally, a specific setting was used to record ten gait cycles per test sequence. This enables to perform a more comprehensive analysis of motion features and their variation.

## 2 Data Description

Dynamic ST is able to reconstruct rotation values for a range of vertebrae recorded over a certain period of time. Thus, ST data, as provided in this article (accessible under [3]), represents a time series, where the particular vertebral orientation is stored over a set of time steps. For determination of rotation values, ST systems project light patterns on the back of a test person and measure the surface structure based on a triangulation method [4]. This yields 3d coordinates of surface points over a broad area of the person's back. Subsequently, the rotational alignment of the vertebrae is indirectly derived from the shape of the surface. More precisely, the local curvature along the spine is used for this purpose [4].

ST are not precisely reflecting orientation values as given by x-ray measurements that are the gold standard for the assessment of static posture of the spine [5]. But, ST provides consistent measurements of spinal posture, which can be utilized e.g. for screening purposes or the surveillance of the progression of diseases like scoliosis [5,6]. In comparison to X-ray measurements with their ionizing radiation, ST systems are not only applicable in static situations. To a certain level of accuracy, ST measurements are able to record vertebral orientation in dynamic situations [7], [8], [9]. Up to now, validity of ST measurements with respect to specific pathologies has only be shown for static measurements [10], but not for dynamic gait analysis. The angular values are not guaranteed to be in precise alignment with the true orientation in the corresponding anatomical coordinate system. However, for gait analysis, reliability and reproducibility of the ST data were demonstrated in terms of a test-retest scenario on the same day [9] and in comparison with a marker based tracking system [8]. Due to the issue with ionizing radiation, the parallel measurement of a gold standard, e.g. in terms of dynamic x-ray measurements is hard to justify.

Despite these limitations, the dynamic ST data provided here are still considered to be of high value for further research in motion analysis. If specific motion features can be represented in a consistent and reproducible way, this may lead to insights about the individual behavior of the spine, even though the data might not be in exact agreement with a gold standard. In particular, this applies to the analysis of intra- and interindividual variation that is based on a relative comparison of the particular motion signals. For this purpose, exact consistency with an external gold standard is not of primary importance. Instead, reproducibility of the measurements is crucial.

For example, Haimerl et al. [1] addressed an analysis of intra- and inter-individual variation using visualization techniques. It was shown, that the average orientation of the vertebrae remains consistent even when one person was walking at different speed levels. The variation between test persons was substantially higher. Further on, Haimerl et al. [2] performed an analysis of intra- and inter-individual variation in a more operationalized approach. First, the average vertebral orientation was separated from the remaining signal parts. Second, a clustering approach was used to fully discriminate all test persons based on these motion characteristics. This demonstrated the high intra- and at the same time limited inter-individual variation of these features, i.e. the average vertebral orientation, which represents some kind of neutral profile of the spine. In general, the data provide a chance to get more insights into the behavior of the spine during motion tasks, including their reproducibility, individuality, and variation of motion patterns.

For this purpose, the measurement of vertebral rotation includes a broad range of vertebra levels, from VP (vertebra prominens) down to L4 as well as the pelvis. L5 is generally omitted by the system since measurements for this vertebra are not guaranteed to be accurate according to the low position of L5 which does not allow a discrimination from the pelvis. The same applies for the remaining part of the cervical spine. Rotational data are recorded in all motion directions, i.e. axial rotation (AxRot), lateral flexion (LatFlex), and flexion/extension (FlexExt). The rotation values are calculated as projection angles in the corresponding direction. For this purpose, the spatial orientation of the room/ST system is used as a global coordinate system. The basic upright posture of the test person is considered to be roughly aligned with this coordinate system. The following definition of rotation directions is used, including assignment to positive and negative values of the motion directions.

AxRot: rotation around the up-down axis of the room (identical to up-down direction for the ST-system), positive sign indicates a rotation to the right for the global parameters (clockwise rotation when seen from back), and a rotation to the left for the specific vertebral body data (counterclockwise rotation when seen from back).

LatFlex: rotation around the front-back axis of the ST-system positive sign reflects rotation to the left (counterclockwise rotation when seen from back)

FlexExt: rotation around the left-right axis of the ST-system positive sign represents flexion, negative sign extension

**Fig 1:**
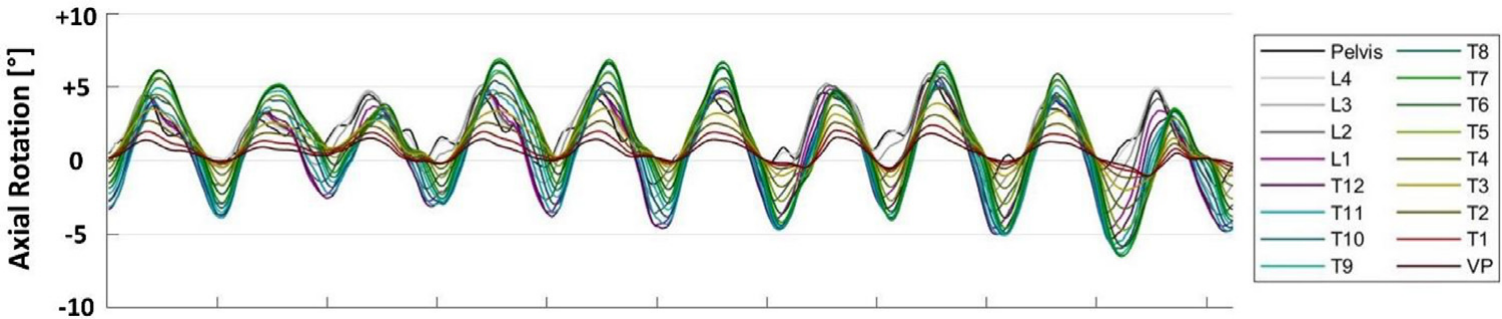
Vertebral rotation data with respect to axial rotation (AxRot) shown as a time series for one test sequence, i.e. one test person walking 10 full gait cycles at a given speed level (here: 3 km/h). The y-axis represents rotation in degree, the x-axis the number of the time step.

**Fig 2:**
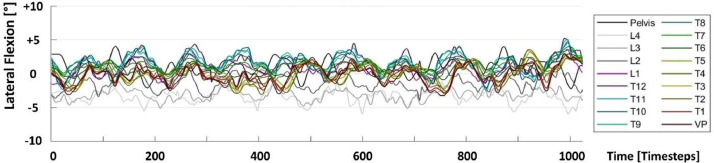
Vertebral rotation data with respect to lateral flexion (LatFlex) shown as a time series for one test sequence. See Fig 1 for more details.

**Fig 3:**
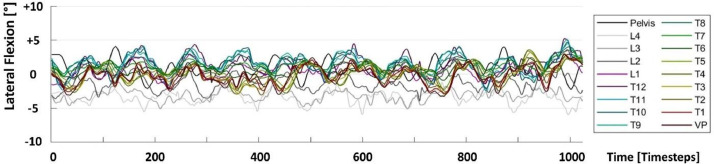
Vertebral rotation data with respect to flexion/extension (FlexExt) shown as a time series for one test sequence. See Fig 1 for more details.

As already mentioned, the rotation data were recorded as a time series. Each single data set reflects a test sequence, where one test person was walking on a treadmill for 10 full gait cycles at a given walking speed (see section ‘experimental design’ for details). Fig 1 provides a graphical representation of the resulting motion signals, in this case for AxRot. The recorded data is represented in superposition of all vertebral levels. A color-coding, as shown in the legend, is used for a better discrimination of the vertebrae. The rotation values are encoded on the y-axis whereas the time steps are shown on the x-axis, containing 10 full gait cycles (each cycle including left and right step). The time series was not normalized to single steps or gait cycles. Thus, the number of time steps varies between the steps / gait cycles as well as the different test sequences. Fig. 2 and Fig. 3 provide the same representation for the other motion directions, i.e. for LetFlex and FlexExt.

The complete data set consists of 36 test sequences. For each individual, three test sequences were recorded where the test person was walking at a predefined speed level (2 km/h, 3 km/h, 4 km/h). Overall, a full set of speed levels were recorded for 11 test persons. For one test person, the speed level was incorrectly assigned in some of the cases. For this test person, one speed level (3 km/h) is not available. Table 1 shows the full set of test sequences. For anonymization purposes, each test person was represented by an ID, from 9001 to 9011. The remaining three IDs, i.e. 9012 – 9014, are all related to the final test person with the incorrectly assigned speed levels. In this case, only two different speed levels were recorded (2 km/h and 4 km/h). The first recordings for this individual were combined in ID 9012. The following to test sequences (ID 9013 and ID 9014) were repetitions of this test, but again only including 2 km/h and 4 km/h.

**Table 1:** Summary of test sequences contained in the data sets, including the IDs for the particular test persons as well as available speed levels.

ID	Included Speed Levels			Comment
9001	2	3	4	
9002	2	3	4	
9003	2	3	4	
9004	2	3	4	
9005	2	3	4	
9006	2	3	4	
9007	2	3	4	
9008	2	3	4	
9009	2	3	4	
9010	2	3	4	
9011	2	3	4	
9012	2		4	All these test sequences are related to the same test person.
9013	2			
9014			4	

The overall data set, including all test sequences, is stored comprehensively in the Excel file ‘RawData_ST gait analysis data_2022-01-27_overall.xlsx’. Additionally, csv files are provided where the data set is split into the particular test sequences. For each test person / ID (e.g. 9002) and speed level (SL), a separate csv file is provided. This information is encoded in the file name as ‘RawData_ST gait analysis data_2022-01-27_ID_SL.txt’ where ID and SL have to be replaced by the corresponding values. For example, this yields ‘RawData_ST gait analysis data_2022-01-27_9002_2.txt’ for test person 9002 and speed level 2 km/h.

The overall Excel file includes the description of the different measurement values in the first lines. This information is omitted in the csv files. In these files, only the pure data values are included. In both cases, each line represents a particular time step, whereas the columns include the various measurements and parameters for this time step. An overview about the contained information can be seen in Table 2. The table includes the initial lines as provided in the overall Excel file. This contains the original description of the data columns in German in the first (grey) line, an English translation in the second (blue) line as well as a column index for better accessing the column information in the third (green) line. These three lines are missing in the csv files for the particular test sequences. Table 3 provides the full set of descriptions of the data columns, which are applicable for the Excel as well as the csv files.

**Table 2:** Basic structure of the provided data – summarized data categories. In particular, this applies to the Excel file ‘RawData_ST gait analysis data_2022-01-27_overall.xlsx’ containing the full data set. Here, the description of the different values is included in the first lines – using the same color coding as in the Excel file. For the csv files, this information is omitted and only the pure data values are contained for a single test sequence. In the table, the white areas represent pure descriptive information, which is not included in the Excel and csv files. The light reddish area is the actual data field. The column indices relevant for the particular category are listed in the green line in order to provide an overview where the particular information can be found.

**Table 3:** Full list of descriptions for the data columns. The listed columns indicate their appearance in the Excel file ‘RawData_ST gait analysis data_2022-01-27_overall.xlsx’. The column index refers to the index number of the column. The following definitions of landmarks are used:








Definitions and images were assigned by DIERS (© Florian Franke / DIERS) and adapted by authors.

### 2.1 Experimental Design, Materials and Methods

The data was acquired within a study (cf. [1]) to assess intra- and interindividual motion characteristics in gait sequences. The data were recorded using the surface topography system DIERS 4D motion® Lab / DIERS formetric 4D system (Version 3.10.1, Diers International GmbH, Schlangenbad, Germany). Surface topography is also known as rasterstereography [8]. The system was combined with a treadmill for ensuring a consistent walking speed. Additionally, the pedogait component was included for identifying the particular steps / gait cycles. The starting and end point of a step was identified according to the contact of the particular foot to the ground. The pedogait component is a 1 meter long capacitive pressure plate with 48 × 112 sensors, which measures the foot pressure reaction forces while walking. The frequency of the camera recordings was 60 Hz and the frequency of the pedogait system 100 Hz.

In total, 12 volunteers, 10 males and 2 females in the age between 20 and 70, participated in the study. Only asymptomatic and pain free volunteers were included, i.e. individuals without self-reported spinal problems. The BMI had to be below 30, since this was a limitation of the used ST system. As an additional inclusion criterion, it was required that the test person was able to walk without problems on the treadmill at each addressed motion speed. All data were stored in a fully anonymized way. No further demographic data was recorded, e.g. mean age or concrete BMI values. All data acquisition steps took place in Nov 2018.

Each individual had to perform test sequences at three different speed levels (i.e. 2, 3, and 4 km/h). For one of the test persons, one speed level was set incorrectly. Based on this, full data sets are only available for 11 individuals. Each test sequence contained 10 full gait cycles including left and right steps. The acquisition time for each test sequence was approximately 20 seconds. Before starting the recordings, the test persons were equipped with the standard measurement equipment of the DIERS 4D motion® Lab system, as defined in the system's manual. Reflective markers were attached to specific anatomical landmarks, including vertebra prominens and both dimples. During the recordings, the test persons only wore underwear. A habituation phase was included to familiarize the test persons with the system. Within the habituation phase, the speed was incrementally increased to prevent tripping. All further measurement steps were performed as specified by the software. For each test person, the recordings took place on a single day. The data was provided as raw data in order to enable a broad range of signal processing and further evaluation steps regarding motion characteristics and their intra- and interindividual variation.

## Ethics Statements

The data presented in this paper was acquired in a fully anonymized way. According to the responsible ethics committee of the medical chamber Rhineland-Palatinate (2021.03.11), there were no objections to process and publish them as anonymous results. Additionally, there were no objections with respect to the Declaration of Helsinki, in general.

## CRediT authorship contribution statement

**Martin Haimerl:** Conceptualization, Methodology, Data curation, Validation, Project administration, Supervision, Writing – original draft. **Alina Linkerhägner:** Conceptualization, Methodology, Investigation, Data curation. **Jürgen Konradi:** Conceptualization, Methodology, Validation, Project administration, Supervision, Writing – review & editing. **Claudia Wolf:** Methodology, Data curation, Validation, Project administration, Writing – review & editing. **Philipp Drees:** Resources, Supervision. **Ulrich Betz:** Conceptualization, Methodology, Supervision.

## Declaration of Competing Interest

The authors declare that they have no known competing financial interests or personal relationships that could have appeared to influence the work reported in this paper. The authors declare the following financial interests/personal relationships which may be considered as potential competing interests: Alina Linkerhägner: The acquisition of the data was performed during her Bachelor thesis at Diers International GmbH, Schlangenbad, Germany. However, this was only related to the acquisition of the data but not to subsequent processing.

## Data Availability

https://data.mendeley.com/datasets/y8nwmwvs5n/1 (Original data) (Mendeley Data).
